# Systematic review and meta-analysis of cell therapy for COVID-19: global clinical trial landscape, published safety/efficacy outcomes, cell product manufacturing and clinical delivery

**DOI:** 10.3389/fimmu.2023.1200180

**Published:** 2023-06-21

**Authors:** Pedro S. Couto, Nada Al-Arawe, Igor S. Filgueiras, Dennyson L. M. Fonseca, Irene Hinterseher, Rusan A. Catar, Raghavan Chinnadurai, Alexey Bersenev, Otávio Cabral-Marques, Guido Moll, Frances Verter

**Affiliations:** ^1^ Department of Biochemical Engineering, Advanced Centre for Biochemical Engineering, University College London, London, United Kingdom; ^2^ CellTrials.org and Parent’s Guide to Cord Blood Foundation, a non-profit organization headquartered in Brookeville, Brookeville, MD, United States; ^3^ Department of Nephrology and Internal Intensive Care Medicine, Charité Universitätsmedizin Berlin, corporate member of Freie Universität Berlin, Humboldt-Universität zu Berlin, Berlin Institute of Health (BIH), Berlin, Germany; ^4^ Vascular Surgery Clinic, Charité Universitätsmedizin Berlin, Berlin, Germany; ^5^ Department of Immunology, Institute of Biomedical Sciences, University of São Paulo (USP), São Paulo, SP, Brazil; ^6^ Interunit Postgraduate Program on Bioinformatics, Institute of Mathematics and Statistics (IME), University of São Paulo (USP), São Paulo, SP, Brazil; ^7^ Department of Vascular Surgery, Universitätsklinikum Ruppin-Brandenburg, Medizinische Hochschule Brandenburg Theodor Fontane, Neuruppin, Germany; ^8^ Fakultät der Gesundheitswissenschaften Brandenburg, Gemeinsame Fakultät der Universität Potsdam, der Medizinischen Hochschule Brandenburg Theodor Fontane, und der Brandenburg Technischen Universität (BTU) Cottbus-Senftenberg, Potsdam, Germany; ^9^ Department of Biomedical Sciences, Mercer University School of Medicine, Savannah, GA, United States; ^10^ Advanced Cell Therapy (ACT) Laboratory, Yale School of Medicine, New Haven, CT, United States; ^11^ Department of Clinical and Toxicological Analyses, School of Pharmaceutical Sciences, University of São Paulo (USP), São Paulo, SP, Brazil; ^12^ Department of Pharmacy and Postgraduate Program of Health and Science, Federal University of Rio Grande do Norte, Natal, Brazil; ^13^ Department of Medicine, Division of Molecular Medicine, University of São Paulo School of Medicine, São Paulo, Brazil; ^14^ Laboratory of Medical Investigation 29, University of São Paulo School of Medicine, São Paulo, Brazil; ^15^ Berlin Institute of Health (BIH) Center for Regenerative Therapies (BCRT), Charité - Universitätsmedizin Berlin, Berlin, Germany

**Keywords:** cell and gene therapy (CGT), advanced therapy medicinal products (ATMPs), mesenchymal stromal/stem cells (MSCs), severe respiratory distress syndrome coronavirus 2 (SARS-CoV2), coronavirus induced disease 2019 (COVID-19)

## Abstract

During the pandemic of severe respiratory distress syndrome coronavirus 2 (SARS-CoV2), many novel therapeutic modalities to treat Coronavirus 2019 induced disease (COVID-19) were explored. This study summarizes 195 clinical trials of advanced cell therapies targeting COVID-19 that were registered over the two years between January 2020 to December 2021. In addition, this work also analyzed the cell manufacturing and clinical delivery experience of 26 trials that published their outcomes by July 2022. Our demographic analysis found the highest number of cell therapy trials for COVID-19 was in United States, China, and Iran (N=53, 43, and 19, respectively), with the highest number per capita in Israel, Spain, Iran, Australia, and Sweden (N=0.641, 0.232, 0,223, 0.194, and 0.192 trials per million inhabitants). The leading cell types were multipotent mesenchymal stromal/stem cells (MSCs), natural killer (NK) cells, and mononuclear cells (MNCs), accounting for 72%, 9%, and 6% of the studies, respectively. There were 24 published clinical trials that reported on infusions of MSCs. A pooled analysis of these MSC studies found that MSCs provide a relative risk reduction for all-cause COVID-19 mortality of RR=0.63 (95% CI 0.46 to 0.85). This result corroborates previously published smaller meta-analyses, which suggested that MSC therapy demonstrated a clinical benefit for COVID-19 patients. The sources of the MSCs used in these studies and their manufacturing and clinical delivery methods were remarkably heterogeneous, with some predominance of perinatal tissue-derived products. Our results highlight the important role that cell therapy products may play as an adjunct therapy in the management of COVID-19 and its related complications, as well as the importance of controlling key manufacturing parameters to ensure comparability between studies. Thus, we support ongoing calls for a global registry of clinical studies with MSC products that could better link cell product manufacturing and delivery methods to clinical outcomes. Although advanced cell therapies may provide an important adjunct treatment for patients affected by COVID-19 in the near future, preventing pathology through vaccination still remains the best protection to date. We conducted a systematic review and meta-analysis of advanced cell therapy clinical trials as potential novel treatment for COVID-19 (resulting from SARS-CoV-2 coronavirus infection), including analysis of the global clinical trial landscape, published safety/efficacy outcomes (RR/OR), and details on cell product manufacturing and clinical delivery. This study had a 2-year observation interval from start of January 2020 to end of December 2021, including a follow-up period until end of July to identify published outcomes, which covers the most vivid period of clinical trial activity, and is also the longest observation period studied until today. In total, we identified 195 registered advanced cell therapy studies for COVID-19, employing 204 individual cell products. Leading registered trial activity was attributed to the USA, China, and Iran. Through the end of July 2022, 26 clinical trials were published, with 24 out of 26 articles employing intravenous infusions (IV) of mesenchymal stromal/stem cell (MSC) products. Most of the published trials were attributed to China and Iran. The cumulative results from the 24 published studies employing infusions of MSCs indicated an improved survival (RR=0.63 with 95% Confidence Interval 0.46 to 0.85). Our study is the most comprehensive systematic review and meta-analysis on cell therapy trials for COVID-19 conducted to date, clearly identifying the USA, China, and Iran as leading advanced cell therapy trial countries for COVID-19, with further strong contributions from Israel, Spain, Australia and Sweden. Although advanced cell therapies may provide an important adjunct treatment for patients affected by COVID-19 in the future, preventing pathology through vaccination remains the best protection.

## Graphical Abstract

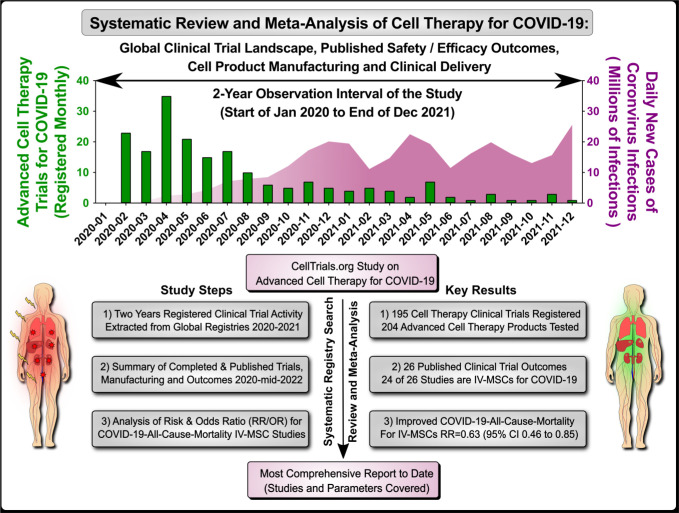

## 1 Introduction

**Figure 1:**
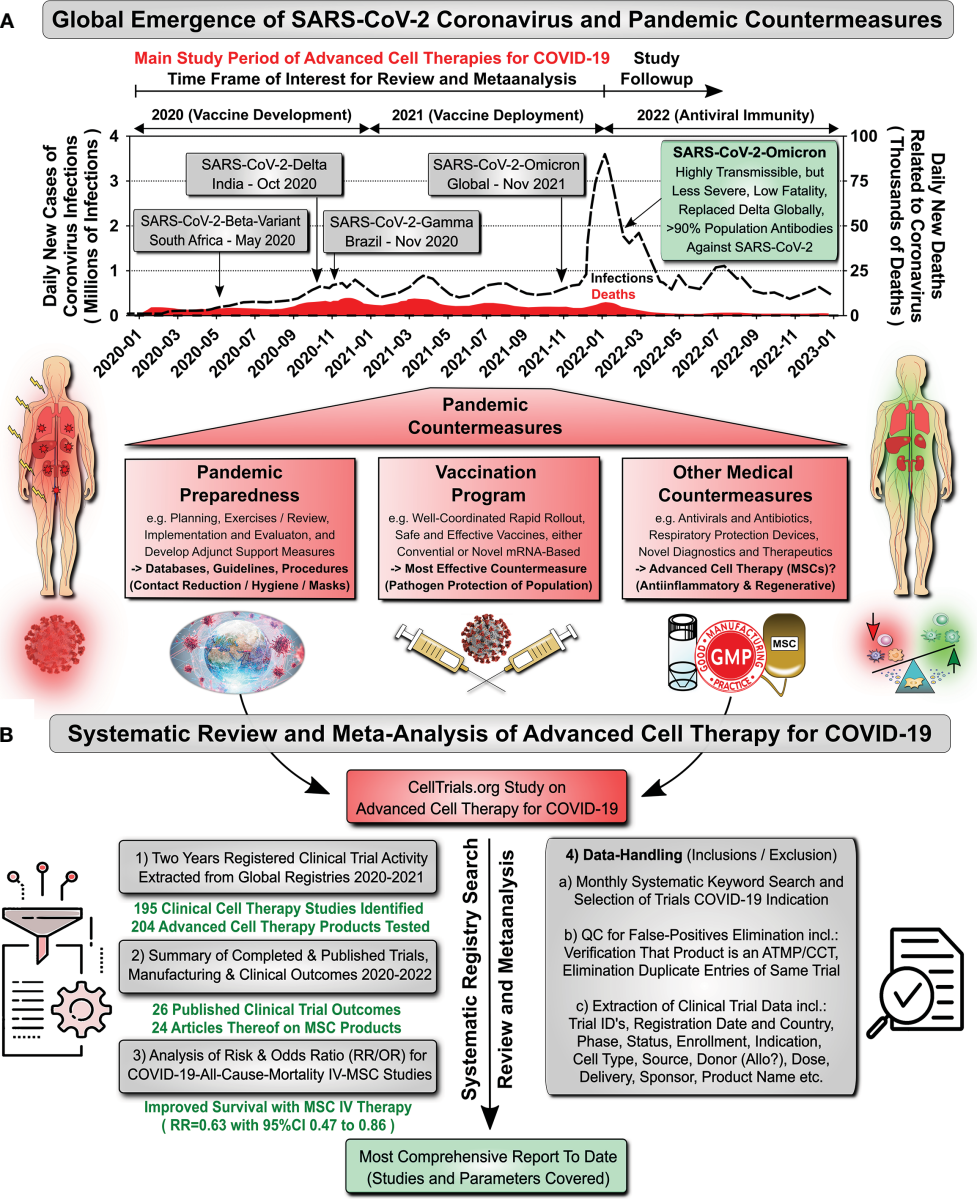
Study Design of Systematic Review & Meta-Analysis of Cell Therapy for COVID-19. **(A)** Global Emergence of SARS-CoV-2 Coronavirus and Pandemic Countermeasures: Data on daily new registered cases of coronavirus infections (Millions of Infections, dotted black curve) and daily new deaths related to coronavirus infection (Thousands of Deaths, red section/curve at bottom) were obtained from the COVID-Worldometer (6). Arrows indicate the emergence of major SARS-CoV-2 variants and interventional measures (2020 start of novel vaccine development programs; 2021 start of novel vaccine deployment, some countries already started in the late fall of 2020, e.g. the US started on the 14^th^ of Dec, Israel on the 20^th^ of Dec, and Germany on 26th of Dec 2020; and 2022 emergence of antiviral immunity in population). Our study period of advanced cellular therapy clinical trials for COVID-19 spans from January 2020 to end of December 2021, with follow-up search for publications until the end of July 2022. Pandemic countermeasures are indicated below the graph, depicting 1) Pandemic Preparedness (e.g. including databases, guidelines, and procedures), 2) Vaccination Programs (most effective countermeasures), and 3) Other Medical Countermeasures (e.g. advanced cell therapies, such as mesenchymal stromal/stem cells, MSCs, of unknown value). **(B)** Systematic Review and Meta-Analysis of Advanced Cell Therapies for COVID-19: Until today most information on advanced cell therapies for COVID-19 is still highly fragmented, in the sense that clinical trials and publications are being compiled separately. This prompted us to conduct this systematic review and meta-analysis to summarize and combine the current knowledge, covering: 1) Two years of registered clinical trial activity, 2) Summary of published clinical trial outcomes, cell product manufacturing and clinical delivery between 2020 and mid-2022, 3) Analysis of risk and odds ratios for COVID-19 all-cause-mortality considering intravenous (IV)-use of MSCs, and 4) A summary of the quality control routines, which were applied for the data-handling (Inclusion/Exclusion) in this study.

The outbreak of the novel severe respiratory distress syndrome coronavirus 2 (SARS-CoV2) and its adjunct symptomatic, Coronavirus 2019 induced disease (COVID-19), is one of the most significant world health events in recorded history (1). Early reports during the initial outbreak in Wuhan, China, found that up to 14% of patients presented with the severe form of COVID-19 and that mortality was as high as 3% (2–4). Subsequently, the virus became a global pandemic and new variants emerged (5–9). Major variants responsible for surges of virus infections include “Beta” (South Africa, May 2020), “Delta” (India, October 2020), and “Gamma” (Brazil, November 2020) (**Figure 1A**) (7). During the summer and fall seasons of 2022, the predominant circulating variants were sub-types of “Omicron”, first documented in November 2021 across multiple countries (10); E.g. the “Omicron” sublineage BQ.1 was designated as a Variant of Interest (VOI) by the European Center for Disease Prevention and Control (ECDC) as of 20^th^ of October 2022 and it was expected that by mid-November to beginning of December 2022 more than 50% of SARS-CoV-2 infections were due to BQ.1/BQ.1.1 (11). This demonstrates the rapid dynamics in virus changes (9). By the end of December 2022, the worldwide death toll attributed directly to COVID-19 had surpassed 6.6 million individuals (5–7).

The COVID-19 pandemic created an ideal situation for the convergence of two research quests that had been progressing independently for decades. The first quest came from pulmonology, where researchers have sought to improve mortality from acute respiratory distress syndrome (ARDS) for decades, with mortality levels of 44% in clinical trials since the 1980’s (12). The second quest, dating back from the 1990s, was the scientific effort to demonstrate clinical efficacy for cell therapy products containing multipotent mesenchymal stromal/stem cells (MSCs) (2, 13–18). Preliminary evidence suggested that MSCs might be beneficial for pulmonary disorders (2, 18). This is supported by biodistribution studies which demonstrated that MSCs given intravenously (IV) rapidly localize to the lungs, where they may exert their beneficial properties (2, 15, 16, 18). It is well established that the immunomodulatory and regenerative properties of MSCs entail a plethora of distinct synergistic mechanisms of action (MoAs) that might help ameliorate pulmonary conditions (13, 18).

Between 2011 to 2019, the database CellTrials.org identified 16 clinical trials of MSCs for ARDS, and by April 2020, seven of these trials were completed, and five were published (19). Unfortunately, none of these publications demonstrated clinical efficacy of MSCs against ARDS. Similarly, a literature search based on published studies of MSCs for ARDS between 1990 to 2020 found nine such studies and confirmed that the improvement in mortality was not significant (20). In the past, poor outcomes considering ARDS mortality were often attributed to the high complexity of ARDS etiology and pathology (e.g. many small/difficult to target subgroups) and rapid disease progression (e.g. short time window for interventional treatment). Thus, any studies aiming to prove efficacy in ARDS typically require stringent inclusion/exclusion criteria and large trial cohorts to control for confounders. This has been challenging to achieve, given that patient enrollment only reaches sufficient numbers in larger specialized clinical centers, thus often requiring multi-center studies (12).

This led to the convergence of the quests mentioned above during the COVID-19 pandemic. For the first-time large cohorts were broadly available to effectively study novel therapeutic interventions. Pulmonologists noted very early that COVID-19 differs from classic presentations of ARDS (21), thus calling for an in-depth analysis of clinical trial outcomes. However, current knowledge on the outcome of cell therapy studies for both ARDS and COVID-19 is still fragmented (**Figure 1B**) (2, 18). Covering the most relevant 2-year interval (Jan 2020 to Dec 2021), we here report the outcomes of the available published clinical trials focusing on cell therapy of COVID-19 today. This also covers specific intricacies of product manufacturing and delivery to patients (2). Our analysis found that three quarters of cell therapies deployed against COVID-19 relied on MSCs. During 2020 alone more than 100 clinical trials were registered worldwide that employed MSCs to treat COVID-19 pneumonia and acute respiratory distress. Indeed, published outcomes from those trials that focus on MSC therapy for COVID-19 now appear to be sufficient to warrant a first comprehensive examination of the safety and efficacy profiles of MSCs for treating severe COVID-19.

## 2 Materials and methods

This study presents three types of data regarding cell-based therapies for COVID-19 (**Figure 1B**): **(1)** We have collected two years (from Jan 2020 to Dec 2021) of registered clinical trial activity extracted from worldwide registries; **(2)** We have gathered the published clinical outcomes and extracted any available information on manufacturing and clinical delivery of MSC products from the published studies to study the potential impact of cell product manufacturing and mode of delivery on clinical efficacy; **(3)** We have performed a relative risk ratio (RR) and odds ratio (OR) analysis for all-cause COVID-19 mortality for studies employing intravenous use of MSCs (most frequent).

### 2.1 Identification of registered clinical trials, keyword search, inclusion/exclusion criteria

The methodology for identifying and assembling the database of clinical trials was the same as described earlier by CellTrials.org (22, 23). Briefly, five main steps were performed monthly: (2.1.1) Keyword-based search (keywords shown below) for advanced cell therapy clinical trials worldwide, (2.1.2) Elimination of false positives, (2.1.3) Elimination of duplicate entries, (2.1.4) Gathering detailed data from included clinical trials, and (2.1.5) Extracting trials from the primary data-base where the indication for cell therapy use was COVID-19. The accuracy of the data search relies on the usage of multiple national registries of clinical trials (**Table 1**), including Australia and New Zealand, Brazil, China, Cuba, Germany, India, Iran, Japan, The Netherlands, Singapore, South Korea, Thailand, the United States of America (USA), the European Union (EU), and World Health Organization (WHO). The time frame for identifying advanced cell therapy trials for COVID-19 was between January 2020 until end of December 2021, containing the large majority of so far registered clinical trials (as detailed below). A tabular excel sheet summary of all 195 identified studies including 20 individual parameters can be found in **Table S1**.

**Table 1:** National Registries Searched for COVID-19 Advanced Cell Therapy Clinical Trials.

Nation	No. of Trials	Registry Name	Registry URL
**Australia & New Zealand**	**4**	**Australian New Zealand Clinical Trial Registry (ANZCTR)**	https://www.anzctr.org.au/
**Brazil**	**2**	**Registro Brasileiro de Ensaios Clinicos (ReBEC)**	https://ensaiosclinicos.gov.br/
**China**	**28**	**Chinese Clinical Trial Registry (ChiCTR)**	http://www.chictr.org.cn/
**Cuba**	**1**	**Registro Público Cubano de Ensayos Clínicos**	https://rpcec.sld.cu/
**EU**	**4**	**EU Clinical Trials Register (EudraCT)**	https://www.clinicaltrialsregister.eu/
**Germany**	**0**	**German Clinical Trials Register (DRKS)**	https://www.drks.de/
**India**	**3**	**Clinical Trials Registry-India (CTRI)**	http://ctri.nic.in/
**Iran**	**18**	**Iranian Registry of Clinical Trials (IRCT)**	https://www.irct.ir/
**Japan**	**1**	**JAPIC Clinical Trials Information**	https://www.clinicaltrials.jp/
**Japan**	**0**	**Japan Medical Association Clinical Trial Registry (JMA-CTR)**	http://www.jmacct.med.or.jp/
**Japan**	**1**	**Japan Registry of Clinical Trials**	https://jrct.niph.go.jp/
**Japan**	**0**	**Japan University hospital Medical Information Network Clinical Trial Registry (UMIN-CTR)**	https://www.umin.ac.jp/ctr/
**Netherlands**	**0**	**Netherlands Trial Register (NTR)**	http://www.trialregister.nl/
**Singapore**	**0**	**Health Sciences Authority Clinical Trial Registry**	https://www.hsa.gov.sg/clinical-trials/clinical-trials-register
**South Korea**	**1**	**Clinical Research Information Service from South Korea (CRiS)**	https://cris.nih.go.kr/
**Thailand**	**0**	**Thai Clinical Trials Registry (TCTR)**	https://www.thaiclinicaltrials.org/
**USA**	**131**	**ClinicalTrials.gov**	https://clinicaltrials.gov
**WHO**	**1**	**World Health Organization International Clinical Trials Registry Platform (ICTRP)**	https://www.who.int/clinical-trials-registry-platform/ **also** https://www.isrctn.com/

This study covers 195 clinical trials worldwide conducting advanced cell therapy for COVID-19 that were registered during the time period Jan. 2020 to Dec. 2021, with a follow-up until end of July 2022 to detect 26 published outcomes of trials.

#### 2.1.1 Keyword search

The keywords used in the first step, broadly designed to capture all advanced cell therapies, included: “COVID-19”, “cell”, “cell therapy”, “cancer vaccine”, “CAR-T”, “chimeric antigen”, “DC”, “NK”, “TIL”, “tumor infiltrating”, “adoptive”, “regenerative”, “mesenchymal”, “adipose”, “bone marrow”, “cord blood”, and “umbilical”.

#### 2.1.2 Elimination of false positives

A second curation step was needed to screen for studies that were performing advanced cell therapy and were not just a false hit on a keyword. We applied the definitions of Advanced Cell Therapy Medicinal Products (ATMPs) adopted by the European Medicines Agency (EMA), and Human Cellular Tissue Products (HCT/Ps) adopted by the US Food and Drug Administration (FDA) (24–26). This step was performed by having at least two scientists review each trial description.

#### 2.1.3 Elimination of duplicate entries

To remove double postings of the same trial in more than one registry, scientific review was applied. If the trial was listed on the US registry ClinicalTrials.gov and another national registry within the same month, then the trial was assigned to ClinicalTrials.gov. If the trial appeared on a second registry months later, it stayed assigned to the month and registry where it first appeared.

#### 2.1.4 Extraction of trial data

The dataset was built by recording the following parameters for each trial: registration date, clinical trial unique ID, secondary ID if any, country of registration, phase, status, cell type, cell source, route of administration, dosage if known, clinical indication, donor type (allogenic or autologous), target enrollment, age ranges of the patient population, type of sponsor (academia or industry), names of the sponsors, and product name if any. (1.5) Selection of trials with COVID-19 indication: The final step for this study was to extract the clinical trials of cell-based therapies where the indication for clinical use was COVID-19. On a monthly basis, we posted them online as an open-access community service. Since the early months of the COVID-19 pandemic, our living database of clinical trials has been listed as a resource on the evidence hub of the Center for Science in the Public Interest (CSPI) (27).

### 2.2 Published clinical trial outcomes, cell therapy manufacturing, and clinical cell delivery

The methodology for gathering information on safety and efficacy from clinical trials of cell-based therapies for COVID-19 has been previously described (23). Briefly, two complementary methods were employed. First, the PubMed registry was searched for publications using the keywords “COVID”, “cell” and “clinical trial”. Second, the search was refined by identifying publications containing the unique ID of each registered trial in our database. We only included publications reporting the outcomes of registered clinical trials but excluded case reports which could not be linked to registered trials. The collected parameters were as follows: connection between trial and publication, country where study was conducted, study design, study endpoints, target enrollment of trial, actual enrollment in paper, cell type(s), cell source(s), cell dose(s), route of administration, adverse events, survival of cell therapy patients and controls. The cut-off date for including publications from trials registered in 2020 and 2021 was the end of July 2022.

#### 2.2.1 World map figures

Global distribution of cell therapy trials for the treatment of COVID-19 per country was displayed as heat map either for the absolute number of trials per country or per capita values. Maps were drawn using the “R” packages ‘maps’ (28) and ‘ggplot2’ (29). The corresponding analysis scripts are available at https://github.com/Starahoush/MSC-COVID19_metaanalysis.

#### 2.2.2 Information on cell therapy manufacturing from published studies

Manufacturing and clinical delivery information for each MSC product were obtained upon close inspection of papers to extract information considering: cell sources, donors, cell isolation, cell expansion, cellular passages, medium formulation, storage (fresh or frozen) (30, 31), and quality control steps (e.g. did MSCs fulfill ISCT minimal criteria)? (17, 32, 33). This search often required checking additional sources when the cell product was supplied by a contract manufacturer or described in an earlier publication.

### 2.3 Risk ratio and odds ratio for COVID-19 all-cause mortality for published studies

Statistical analysis was carried out in “R” version 4.2.1 (34), the meta package version 6.0.0, and visualized *via* forest plots from the same package (35). The Risk Ratio (RR) and the Odds Ratio (OR) were calculated by using the Mantel-Haenszel test (36) and employed to analyze the effect of MSC therapy on the risk/odds of death following COVID-19 infection. The 95% confidence interval for both ratios and the combined statistics are reported for each study. The code used for this analysis was uploaded to https://github.com/Starahoush/MSC-COVID19_metaanalysis.

#### 2.3.1 Handling of missing data

The RR and OR calculations require input studies to have two arms, one of the patients undergoing experimental treatment versus a second arm of control/placebo patients. However, during the COVID-19 pandemic, many clinical trials were conducted that only treated patients and did not have a control group. To incorporate their published outcomes, the following methodology was used to integrate single-arm studies, which lacked control data. First, the mean and median data of the controlled studies were obtained. Then, two assumptions were made about the studies without controls: First, it was assumed that the mean behavior of the missing controls was the same as that in the controlled studies, and second, it was assumed that the ratio of MSC patient number to control patient number was the same as the median for the controlled studies. With these two assumptions it was possible to incorporate the experimental results from single-arm studies to calculate RR and OR for all studies as a group. Of the 24 published MSC studies, 17 had complete information regarding the number of participants and events for both treatment and control groups (37–53); whereas seven studies were missing data for the control group (54–60). Also, three studies (43, 46, 49) were double-arm-zero-event, and we employed a treatment arm continuity correction (TACC) to incorporate them, since otherwise risk/odds ratios could not have been calculated (61, 62).

## 3 Results

### 3.1 2-Year registered global clinical trial landscape of advanced cell therapies for COVID-19

**Figure 2:**
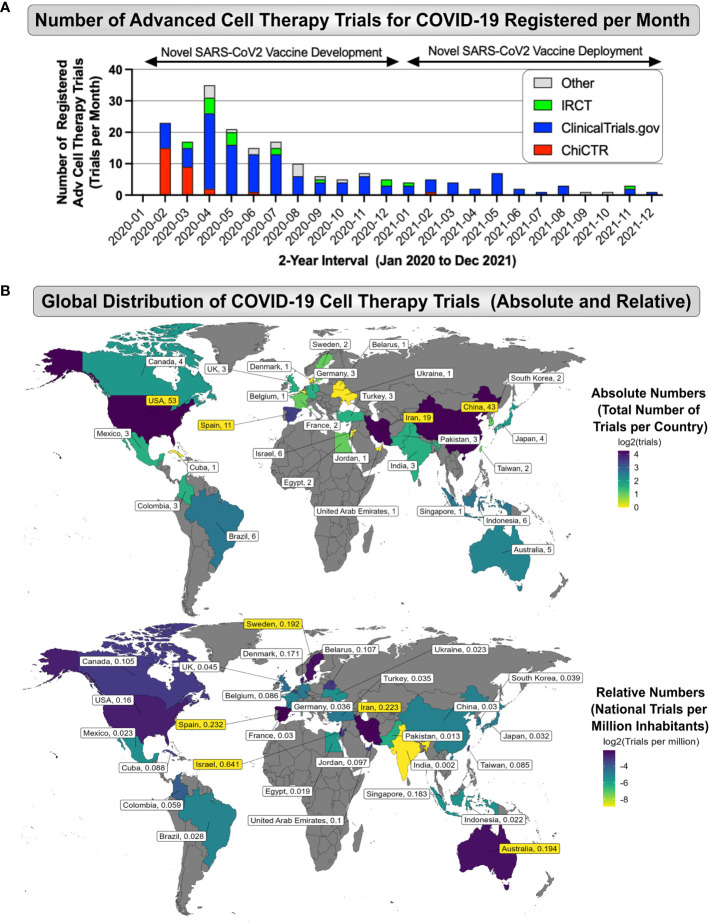
Global Landscape of Cell Therapy Trials for COVID-19. **(A)** Number of Advanced Cell Therapy Trials for COVID-19 Registered per Month: Data on the number of registered advanced cell therapy trials (Trials per Month; depicted is the 2-year interval of interest from the start of January 2020 to end of December 2021) were collected from available national and international clinical trial registries, e.g. the American registry (ClinicalTrials.gov; shown in blue), Chinese registry (ChiCTR, shown in red), Iranian registry (IRCT, shown in green), and other registries (shown in grey), depicting a peak in advanced cell therapy trial registrations between February to July of 2020, with a subsequent long tail of decline in cell therapy trial activity, which occurred at the same time the novel SARS-CoV-2 vaccines showed first success in early clinical trials and started to be deployed (e.g. vector or mRNA-based vaccines); and **(B)** Global Distribution of COVID-19 Advanced Cell Therapy Trials (Absolute and Relative No): The top panel depicts the Absolute Numbers (Total Number of Trials per Country) identifying the US (n=53), China (n=43), Iran (n=19), and Spain (n=11), as the most active countries considering the total trial number output, while the bottom panel depicts the Relative Trial Numbers (National Trials per Million Inhabitants) identifying Israel, Spain, Iran, Australia, and Sweden (N=0.641, 0.232, 0,223, 0.194, and 0.192 trials per million inhabitants) as the most prolific countries relative to their (smaller) national population size, again depicting Iran in place three as for the total trial output.

A comprehensive search for advanced cell therapy trials to target the clinical indication COVID-19 and related complications was conducted in 18 national and international registries (**Table 1**). The cell therapy products employed in these trials are referred to as advanced therapy medicinal products (ATMPs) or human cellular and tissue products (HCT/Ps) in the EU and US, respectively (2, 24, 26). Between January 2020 and December 2021, 195 advanced-cell-therapy-based clinical trials targeting COVID-19 and related complication were registered worldwide (**Table S1**). While we have released first smaller compilations in June 2020 and 2021 (63, 64), the current study covers the most relevant 2-year time window from January 2020 to the end of December 2021 (**Figures 1A**, **2A**). The relevance of this time frame is depicted by the initial peak of monthly registered clinical trials in the first months of the pandemic, followed by dramatic decline and a long tail afterwards. The first clinical trial registrations appeared in China and USA in February 2020. Some February trials were registered retrospectively, but subsequent publications revealed that patients began receiving cell therapy for COVID-19 as early as January 2020 in China. The peak of registrations was April 2020. Noteworthy, registrations of cell therapy trials for COVID-19 had only one peak in spring 2020, although global COVID-19 cases went through four major surges during the 2-year timeframe (5–7). The peak in trial registrations subsided months before the roll out of vaccination programs (65).

To date, information on advanced cell therapy trials for COVID-19 remains fragmented, although first valuable literature reviews and meta-analyses have been conducted, this is the first study that comprehensively connects trials to subsequent publications. We have listed a summary of prior compilations in descending order of the cut-off date of their conducted search (**Table 2**) (63, 64, 66–86). Reviews of COVID-19 clinical trials not strictly focused on cell therapy were excluded from the list. Importantly, the 195 trials identified in this article are more than double the number presented by previous authors, which demonstrates the outreach of our data search criteria. Our compilation of cell therapy trials for COVID-19 is so far the only one that offers worldwide trial data versus time for a 2-year time frame (**Figure 2A**). Our review also tracks contributions of different clinical trial registries over time, illustrated by the respective color coding, which indicates a dominance of contributions from the US (blue), Chinese (red), and Iranian (green) registries, while the contributions from other registries (grey) were smaller. This is partly because the most dominant US registry (clinical.trials.gov, 131 of the 195 registered trials) was used as the default template in our search. It must be noted, that in some countries that have a clinical trial registry, researchers are obligated to use their national registry, and cross posting their trial to ClinicalTrials.gov is optional, so that ClinicalTrials.gov should never be relied upon as a complete international record of clinical trials.

**Table 2:** Previous Compilations of Cell Therapy Trials for COVID-19 Sorted by Search-End-Date.

No. of Trials	Search End	Registries Searched	Authors and Reference
**195 (141 MSC)**	**2021-12**	**ALL (WHO)**	**Couto et al., 2023 (this article)**
**82 MSC**	**2021-10**	**ClinicalTrials.gov**	**Grumet Sherman Dorf 2022 (** 66 **)**
**51 MSC**	**2021-10**	**ClinicalTrials.gov & ChiCTR**	**Lu et al., 2022 (** 67 **)**
**185 (134 MSC)**	**2021-06**	**ALL (WHO)**	**Verter & Couto 2021 (** 64 **)**
**89**	**2020-12**	**ClinicalTrials.gov**	**Zaki et al., 2021 (** 68 **)**
**22 MSC**	**2020-11**	**WHO**	**Khoury et al., 2021 (** 69 **)**
**88**	**2020-08**	**WHO**	**Li et al., 2020 (** 70 **)**
**79**	**2020-08**	**ClinicalTrials.gov & ChiCTR**	**Kim & Knoepfler 2021 (** 71 **)**
**71**	**not stated**	**ClinicalTrials.gov**	**Golchin 2021 (** 72 **)**
**111 (85 MSC)**	**2020-06**	**WHO**	**Verter & Couto 2020 (** 63 **)**
**57**	**2020-06**	**ClinicalTrials.gov & ChiCTR**	**Choudhery & Harris 2020 (** 73 **)**
**54 MSC**	**2020-06**	**ClinicalTrials.gov**	**Shetty et al., 2021 (** 74 **)**
**4 NK**	**2020-05**	**ClinicalTrials.gov**	**Market et al., 2020 (** 75 **)**
**61**	**2020-04**	**PubMed & Cochrane**	**Rada, Corbalán, Rojas 2020 (** 76 **)**
**54**	**2020-04**	**ClinicalTrials.gov & WHO**	**Liao et al., 2020 (** 77 **)**
**29 MSC**	**2020-04**	**ClinicalTrials.gov & ChiCTR**	**Sahu, Siddiqui, Cerny 2021 (** 78 **)**
**28 MSC**	**2020-04**	**ClinicalTrials.gov & WHO**	**Zumla et al., 2020 (** 79 **)**
**16**	**2020-04**	**WHO**	**Thorlund et al., 2020 (** 80 **)**
**15**	**2020-04**	**ClinicalTrials.gov**	**Babaei et al., 2020 (** 81 **)**
**31**	**2020-03**	**ClinicalTrials.gov & ChiCTR**	**Golchin et al., 2020 (** 82 **)**
**23**	**2020-03**	**ClinicalTrials.gov & ChiCTR**	**Khoury et al., 2020 (** 83 **)**
**24 MSC**	**2020-03**	**WHO**	**Ji, Liu, Zhao 2020 (** 84 **)**
**24 MSC**	**2020-03**	**WHO**	**Lythgoe & Middleton 2020 (** 85 **)**
**5 MSC**	**2020-02**	**ClinicalTrials.gov**	**Liu et al., 2020 (** 86 **)**

Previous published compilations of clinical trials conducting cell therapy for COVID-19. Publications are sorted according to the cut-off date of trial collection. **Abbreviations:** WHO, world health organization; and ChiCTR, Chinese clinical trials registry.

A global heatmap of the countries where clinical trials of cell therapy for COVID-19 were conducted, regardless of where they were registered, is shown in **Figure 2B**. Only one trial took place in more than one country. Among 30 participating countries, leaders were the US (n=53, 27%), China (n=43, 22%), Iran (n=19, 10%), and Spain (11, 6%), while other countries hosted <4% (**Figure 2B** **top**). The highest relative trial numbers per capita came from Israel, Spain, Iran, Australia, and Sweden (N=0.641, 0.232, 0,223, 0.194, 0.192 trials/million inhabitants, respectively) (**Figure 2B** bottom). Our compilation is the only one that identifies Iran as the 3^rd^ absolute and relative leading contributor. Presumably, this is because most trials in Iran are only listed on the Iranian national registry, and not cross-posted to ClinicalTrials.gov. Noteworthy, the list of countries leading in cell therapy for COVID-19 through the end of 2021 (US, China, and Iran) does not match the lists of countries that reported the highest number of COVID-19 infections (US, India, France, Brazil) or the highest number of COVID-19 related deaths (US, Brazil, India, Russia) during that timeframe (6).

### 3.2 Types of cell products in registered clinical trials

**Figure 3:**
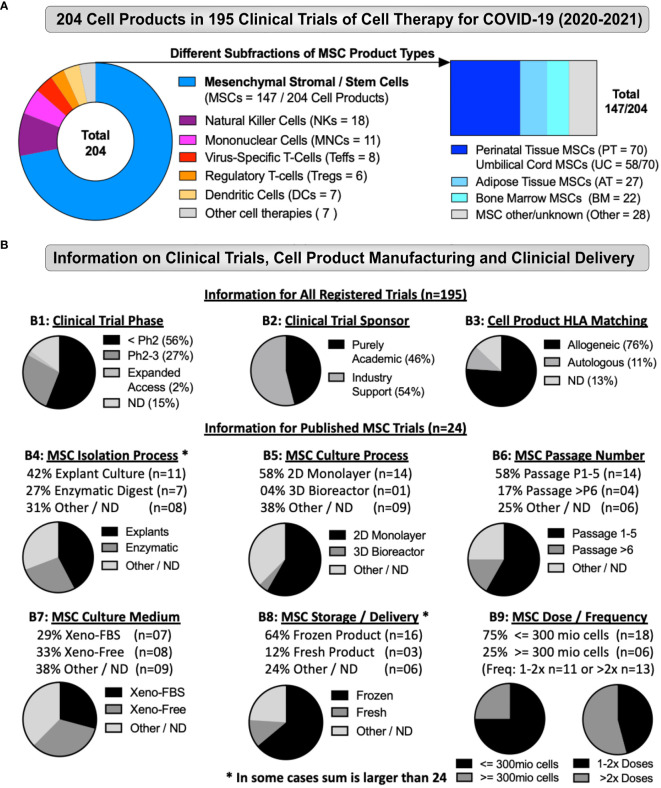
Cell Types and Manufacturing in Cell Therapy Trials for COVID-19. **(A)** Different Types of Cell Products Tested as Therapy for COVID-19 (2020-2021): The figure depicts the total number of advanced cell therapy products tested as treatment for COVID-19 in clinical trials registered between the start of January 2020 to end of December 2021 (The number of 204 products depicted here is higher than the 195 trials identified in total, due to the testing of multiple products in some studies). The products tested included several hematopoietic cell types (e.g. Natural Killer cells, virus-specific and regulatory T cells, but also mononuclear, dendritic cells, and others types of products), and in particular mesenchymal stromal/stem cells (MSCs), accounted for 147/204 products (left panel), including different subfractions of MSC product types (right panel; e.g. adipose tissue (AT-MSC; 27/147), bone marrow (BM-MSC; 22/147), and perinatal tissue (PT)-derived cells (70/147), with the latter being most abundant, which was mostly accounted for by the large number of umbilical cord (UC)-derived MSCs (58/147) products tested. **(B)** Clinical Trial Information, Cell Product Manufacturing and Clinical Delivery: (B1-3) Depicts information for all registered trials (n=195), including (B1) Clinical trial phase, (B2) Clinical trial sponsor, and (B3) Cell product HLA-matching, while (B4-9) Depicts information for the published MSC trials (n=24), including (B4) MSC isolation process, (B5) MSC culture process, (B6) MSC passage number, (B7) MSC culture media, (B8) MSC storage and clinical delivery, (B9) MSC dose and dosing frequency. Some displays in (B4-9) are marked with a star (*) to indicate that numbers can be greater than 24, as some published trials employed more than one method or product. Altogether, our analysis depicts a strong dominance in the data set for early stage trials (56%), employing allogeneic MSC products (76%), which were isolated by explant method (42%), cultured in 2D monolayer flasks (58%), expanded up to passage 5 (58%), and delivered intravenously (100%), mainly as cryostorage-derived freeze-thawed product (64%), and dosed below 3 million cells/kg (75%), in either one to two (46%), or even several more doses (54%), most likely to increase the total number of applied therapeutic cells over a given time frame, without increasing the individual therapeutic doses above a limit of 3 million cells/kg. Interestingly, some studies (17%) employed MSCs expanded above passage 5 up to passage 12. Many MSC products were applied thawed shortly upon retrieval from cryostorage as frozen cells (64%), which has previously been shown to compromise the therapeutic properties of clinical MSC products and may even have accounted for earlier trial failures (2, 14, 16–18, 30, 33, 87–91).

Detailed information about the 195 advanced cell therapy trials for COVID-19 registered 2020-2021, including up to 20 individual parameters for each registered trial (listed in the Methods) are listed in **Table S1** with representative plots of important parameters shown in **Figure 3A** and the top of 3B. While the terminology of our database uses “route of administration” and “cell storage”, in the discussion these topics are combined as “clinical delivery”.Among the 195 registered cell therapy trials for COVID-19, most (n=141, 72%) tested some type of MSC product (**Figure 3A**). The next most common cell types were natural killer (NK) cells and mononuclear cells (MNC), employed in 9% and 6% of the trials, respectively. Interestingly, n=7 of the registered trials used more than one cell type, including more than one MSC type, which is why the total number of cell products is n=204 in the chart (**Figure 3A**, left panel). Even with all cell types counted individually MSC-product-based trials still accounted for 147/204 (72%) of the registered cell therapy trials. The most common type of MSC source was perinatal tissue (PT)-derived PT-MSCs, accounting for 70 trials (34% of all cell types, or 48% of MSC products in trials) (**Figure 3A** right panel). Within this category we included umbilical cord (UC)-derived UC-MSCs in 58 trials, or other perinatal sources in 12 trials. This was followed by adipose tissue (AT)-derived AT-MSCs in 27 studies (13% of cell types in trials, or 18% of the MSC products), and bone marrow (BM)-derived BM-MSCs in 22 studies (11% of all cell types in trials, or 15% of the MSC products), and other types of MSC sources in 28 trials (14% of all cell types, and 19% of the MSC products).

Most of the registered trials were early phase research, with at least 56% below phase 2 (**Figure 3B1** and **Table S1**), which is probably an underestimate of early phase trials, since 15% of trials were of unknown phase. There were four phase 3 trials and four trials registered with US FDA under Expanded Access programs. The sponsors of the registered clinical trials were exclusively academic for 46% of the trials (**Figure 3B2**), but the remaining 54% of trials had industry support, typically from the company that manufactured the cell therapy product used in the trial. The large majority, 76% of all registered trials indicated an allogeneic product (**Figure 3B3** and **Table S1**), while only 11% were autologous and 13% of trials did not report or define this aspect. The rationale behind the predominant use of (allogeneic) donor cells and off the shelf products is probably that the enrolled COVID-19 patients were often critically ill, and either unable to provide autologous cells (patient derived), or unable to wait for the autologous product to be manufactured.

### 3.3 Types of cell products in published outcomes of clinical trials

Our search to match clinical trials with reports of their outcomes identified 26 peer reviewed papers accepted for publication by the end of July 2022 (**Table 3**) (37–60, 92, 93). We also included a clinical trial of MSCs for ARDS that was originally registered in 2017 and published in 2021 (57). The study was included, since during the pandemic, the researchers pivoted to conduct a study of MSCs for ARDS induced by COVID-19 (38). Thus, the trial met our inclusion criteria as a published outcome of a registered clinical trial. As stated in the Methods, we excluded publications that could not be associated with registered trials, such as an extensive report on 210 patients that were treated under the approval from the Ministry of Health in Turkey (94), but not registered as a clinical trial. Our database of published trial outcomes includes two pairs of papers from two research groups in China, where in each case the group initially published a safety study (45, 53) and later published their data in a controlled trial (46, 49), respectively. The therapeutic modality reported across the published clinical trials was overwhelmingly allogeneic in all but one of the 26 published trials (96%). This confirms that those allogeneic products favored completion of trials with subsequent publication, while virtually no trials with autologous products were reported within the time frame of our database. Interestingly, many patients received cells from individual (HLA disparate) donors during each infusion in 13 of the 26 published studies (**Table 4**), while two studies used banks of pooled donors, but in 11 studies the donor selection is unknown. This frequent use of allogeneic products raises the issue of potential alloimmune-cross-reactivity from multiple infusions of HLA-mismatched cell products, which should be followed up in more detail in future studies. However, given the widely postulated hypoimmunogenic or immune-privileged status of MSCs, or better said the “immune-evasive nature of MSCs” (95), this critical aspect in clinical cell transplantation appeared to be of less concern in clinical trial design.

**Table 3:** Published Cell Therapy Trials for COVID-19 with Details on Manufacturing.

Trial	Publication Reference	Country	Enrollment Target (Actual)	Principle Cell Type	Cell Dose and Delivery	Manufacturer (Product Name, if any)	Cell Product Manufacturing Method	Quality Control Characterization
NCT04392778	Adas et al., 2021 (37)	Turkey	30 (30)	UC-derived PT-MSCs	3 x 3.0 million cells/kg IV	Liv MedCell, Liv Hospital	Donors: Individuals; Cell isolation: Explants; Expansion: ND; Passage: P7; Medium: ND; and Storage: Frozen	Immunophenotype, Sterility, and Viability
IRCT20200621047859N4	Aghayan et al., 2022 (38)	Iran	20 (20)	Placenta-derived PT-MSCs	1 x 1.0 million cells/kg IV	Motamed Cancer Institute, Tehran Univ of Medicine	Donors: Individuals/C-section; Cell isolation: Enzymatic; Expansion: Monolayer; Passage: P6/P7; Medium: Xeno-Free; Storage: Frozen	Immunophenotype, Trilineage Differentiation, Sterility, Karyotype, and Viability
NCT04457609	Dilogo et al., 2021 (39)	Indonesia	40 (40)	UC-derived PT-MSCs	1 x 1.0 million cells/kg IV	Stem Cells Medical Technology, Cipto Mangunkusumo Hospital	Donors: ND; Cell Isolation: Explants; Expansion: ND; Passage: P6/P7; Medium: ND; and Storage: ND	Immunophenotype
IRCT20160809029275N1	Farkhad et al., 2022 (40)	Iran	20 (20)	UC-derived PT-MSCs	3 x 1.0 million cells/kg IV	Mashhad University	Donors: Individuals; Cell Isolation: Enzymatic; Expansion: Monolayer; Passage: P3; Medium: α-MEM + 20% FBS; and Storage: Fresh	Immunophenotype, Trilineage Differentiation, Sterility, and Viability
NCT04269525	Feng et al., 2020 (54)	China	16 (16)	UC-derived PT-MSCs	4 x 100 million cells IV	Jilin Tuoha Biotech	Donors: ND; Cell Isolation: ND; Expansion: ND; Passage: ND; Medium: ND; Storage/Use: ND	ND
NCT04445454	Gregoire et al., 2022 (41)	Belgium	20 (32)	BM-derived BM-MSCs	3 x 1.5-3.0 million cells/kg IV	University of Liège	Donors: Individuals; Cell Isolation: Adherence; Expansion: Monolayer; Passage: P3; Medium: DMEM + 10% FBS; and Storage: Frozen	Immunophenotype, Morphology, Karyotype, Viability, Immunosuppression
NCT04377334	Häberle et al., 2021 (42)	Germany	40 (23)	BM-derived BM-MSCs	2-3 x 1.0 million cells/kg IV	Medac (MSC-FFM, aka Obnitix)	Donors: 8 pooled; Cell Isolation: Sepax and Adherence; Expansion: Monolayer/Quantum Bioreactor; Passage: P3; Medium: DMEM + 10% hPL; and Storage: Frozen	Immunophenotype, Sterility Viability
IRCT20200217046526N2	Hashemian et al., 2021 (55)	Iran	6 (11)	UC-derived vs Placenta-derived PT-MSCs	3 x 200 million cells IV	Royan Institute	Donors: Individuals/Vaginal Delivery; Cell Isolation: UC Enzymatic vs Placenta Explants; Expansion: UC ND vs Placenta Monolayer; Passage: UC-MSCs P4 vs Placenta ND; Medium: DMEM + 10% FBS; Storage: UC-MSCs Frozen vs Placenta MSCs Fresh	Immunophenotype, and Viability
NCT04416139	Iglesias et al., 2021 (56)	Mexico	10 (5)	UC-derived PT-MSCs	1 x 1.0 million cells/kg IV	CBCells Biotech	Donors: ND; Cell Isolation: ND; Expansion: ND; Passage: ND; Medium: ND; Storage: ND	ND
NCT04535856	Karyana et al., 2022 (43)	Indonesia	9 (9)	Embryonic-Cell-derived MSCs	50-100 million cells IV	National Institute of Health, South Korea	Donors: Fetus; Cell Isolation: eSC-line; Expansion: Monolayer; Passage: P12; Medium: Xeno-free; and Storage: Frozen	Immunophenotype, Sterility, Trilineage Differentiation, and Tumorigenesis
NCT04355728	Lanzoni et al., 2021 (44 )	USA	24 (24)	UC-derived PT-MSCs Sub-Epithelial	2 x 100 million cells IV	Therapeutic Solutions International (JadiCell)	Donors: Individuals; Cell isolation: Explants; Expansion: Monolayer; Passage: P3; Medium: α-MEM + 10% hPL; and Storage: Frozen	Immunophenotype, Sterility, Viability, and Trilineage Differentiation
ChiCTR2000029990	Leng et al., 2020 (45)	China	120 (10)	UC-derived PT-MSCs ACE2neg	1 x 1.0 million cells/kg IV	Qingdao Co-orient Watson Biotech Group	Donors: ND; Cell Isolation: ND; Expansion: ND; Passage: P3; Medium: DMEM + 2% FBS; and Storage: Frozen	Immunophenotype, Viability, and Trilineage Differentiation
NCT04252118	Meng et al., 2020 (46)	China	20 (9)	UC-derived PT-MSCs	3 x 30 million cells IV	Vcanbio Cell & Gene Engineering	Donors: ND; Cell Isolation: Explants; Expansion: Monolayer; Passage: P5; Medium: Serum-Free; and Storage: ND	Morphology, Immunophenotype, and Trilineage Differentiation
NCT04333368	Monsel et al., 2022 (47)	France	40 (45)	UC-derived PT-MSCs	3 x 1.0 million cells/kg IV	Saint-Louis Hospital Cell Therapy Unit	Donors: Individuals; Cell Isolation: Enzymatic or Explants; Expansion: ND; Passage: P4; Medium: Nutristem^®^ MSC XF + 5% hPL; and Storage: Frozen	Immunophenotype, Sterility, Viability, Karyotype, T-cell-Inhibition Proliferation Assay
NCT04578210	Pérez-Martínez et al., 2021 (92)	Spain	58 (9)	Memory T-cells (CD45RA-)	1 x 0.1, 0.5, 1.0 million cells/kg IV	Hospital La Paz	Donors: Convalescent donors 1 HLA match with patient; Cell Isolation: CliniMacsPlus; Expansion: Monolayer; Passage: P12; Medium: SF/XF; and Storage: Frozen	Immunophenotype and Viability
U1111-1254-9819	Rebelatto et al., 2022 (48)	Brazil	15 (17)	UC-derived PT-MSCs	3 x 0.5 million cells/kg IV	Pontifícia Universidade Católica do Paraná	Donors: Individuals/C-section; Cell Isolation: Enzymatic; Expansion: Monolayer; Passage: P3; Medium: IMDM + 20% FBS; and Storage: Frozen	Immunophenotype, Sterility, and Trilineage Differentiation
IRCT2017010531786N1	Sadeghi et al., 2021 (57)	Iran	15 (10)	Placenta Decidua-derived MSCs	1-2 x 1.0 million cells/kg IV	Taleghani Hospital (DSCs)	Donors: Individual/C-section; Isolation: Enzymatic; Expansion: Monolayer; Passage: P4/P5; Medium: ND; and Storage/Use: Frozen	Immunophenotype, Viability, and Karyotyping
IRCT20190717044241N2	Saleh et al., 2021 (58)	Iran	5 (5)	UC-derived PT-MSCs	3 x 150 million cells IV	CellThecPharmed	Donors: Individuals; Cell Isolation: Explants; Expansion: Monolayer; Passage: P5; Medium: ND; and Storage: Fresh	Immunophenotype, and Trilineage Differentiation
2020-001266-11	Sanchez-Guijo et al., 2020 (59)	Spain	100 (13)	AT-derived AT-MSCs (Liposuction)	1-3 x 1.0 million cells/kg IV	Hospitals Salamanca, Navara, Gregorio Marañón	Donors: Individuals Liposuction; Cell Isolation: Enzymatic; Expansion: Monolayer; Passage: ND; Medium: DMEM + 10% FBS; and Storage: Frozen	Morphology, Immunophenotype, Viability, and Trilineage Differentiation
CTRI/2020/08/027043	Sharma et al., 2022 (60)	India	20 (10)	UC- and Placenta-derived PT-MSCs	2 x 100 million cells IV	ReeLabs	Donors: ND; Cell Isolation: ND; Expansion: ND; Passage: ND; Medium: StemPro MSC SFM/XF medium; and Storage: ND	ND
NCT04288102	Shi et al., 2021 (49 )	China	100 (100)	UC-derived PT-MSCs	3 x 40 million cells IV	Vcanbio Cell & Gene Engineering	Donors: ND; Cell Isolation: Explants; Expansion: Monolayer; Passage: P5; Medium: Serum-Free; and Storage: ND	Morphology, immunophenotype and Trilineage Differentiation
ChiCTR2000031494	Shu et al., 2020 (50 )	China	36 (41)	UC-derived PT-MSCs	1 x 2.0 million cells/kg IV	Jiangsu Cell Tech Biotech	Donors: ND; Cell Isolation: ND; Expansion: ND; Passage: P3-P5; Medium: ND; and Storage: ND	Immunophenotype
NCT04338347	Singh et al., 2020 (51)	USA	Unknown (6)	Cardiac-derived Stromal Cells	1-2 x 150 million cells IV	Capricor (CAP-1002)	Donors: Cadaveric Donor Biopsy; Cell Isolation: Explants; Expansion: Monolayer; Passage: ND; Medium: ND; and Storage: Frozen	Immunophenotype
NCT04473170	Ventura-Carmenate et al., 2021 (93)	UAE	146 (139)	Peripheral Blood-derived Non-Hematopoietic Cells	1 x 2.2 million cells *via* Nebulizer	Abu Dhabi Stem Cells Center	Donors: Autologous; Cell Isolation: Centrifugation; Expansion: ND; Passage: ND; Medium: ND; and Storage: Fresh	Immunophenotype, and Viability
ChiCTR2000029606	Xu et al., 2021 (52)	China	63 (40)	Menstrual Blood-derived MSCs	3 x 30 million cells IV	Innovative Precision Medicine	Donors: Three Individuals; Cell Isolation: Ficoll-Paque Gradient; Expansion: Monolayer; Passage: ND; Medium: ND; and Storage: Frozen	Immunophenotype, Viability, Trilineage Differentiation
NCT04339660	Zhu et al., 2021 (53)	China	30 (58)	UC-derived PT-MSCs ACE2neg	1 x 1.0 million cells/kg IV	Qingdao Co-orient Watson Biotech Group	Donors: ND; Cell Isolation: ND; Expansion: ND; Passage: P3; Medium: DMEM+ 2% FBS; and Storage: Frozen.	Immunophenotype, Viability; Trilineage Differentiation

Publications from completed clinical trials of advanced cell therapy for COVID-19 that were registered Jan. 2020 to Dec. 2021 and published by the end of July 2022. The 26 publications are listed alphabetically by the first author. MSCs, mesenchymal stromal/stem cells; ND, not detailed or not determined; AT, adipose tissue; BM, bone marrow; PT, perinatal tissue; and UC, umbilical cord.

**Table 4:** Summary of Key Manufacturing Parameters from all 26 Published COVID19 Trials.

Manufacturing Parameter	Option 1	Option 2	ND/Other
**Manufacturer**	18 Commercial Labs	8 Academic Labs	0 ND
**Donors**	13 Individuals	2 Pooled	11 ND
**Cell Isolation**	11 Explants	7 Enzymatic	7 ND/3 Other
**Cell Expansion**	15 Monolayer	1 Bioreactor	9 ND/1 None
**Cell Passage**	1 None	19 report P3 – P12	6 ND
**Cell Medium**	7 Xenogenic	9 Xeno-free	9 ND/1 None
**Cell Storage**	4 Fresh	17 Frozen	6 ND

Summary of cell manufacturing parameters in 26 publications from completed cell therapy trials for COVID-19 registered Jan. 2020 to Dec. 2021 and published by the end of July 2022. ND, not detailed or not determined.

By our count, 18 of 26 published trials used cell products from a commercial entity (**Table 4**). Examples are proprietary cell product under development, or cells manufactured by a contract manufacturing organization (CMO), a biotech spin-off, or a cell therapy clinic. By comparison, eight studies used cells manufactured in the lab of an academic center, such as a university lab or a research hospital. This split between commercial facilities *versus* academic labs strongly impacts manufacturing data reporting. In the case of academic labs, manufacturing details are often available, but frequently buried in a supplement of the COVID-19 study, or in a previous paper. When authors used commercial facilities, they often did not describe cell manufacturing, simply citing that the cell products were approved for clinical use by their government. Some of the commercial entities that provided cells for COVID-19 trials have never described their cell manufacturing in any publication, so it is impossible for a reader to know how the cell product was produced and characterized. Given that MSCs were the dominant cell product in the registered cell therapy trials for COVID-19 (72% of all registered trials), it is not surprising that MSC products were employed in 24/26 (92%) of the published clinical trials, while the remaining two employed memory T-cells from convalescent donor plasma (92), or non-hematopoietic cells from peripheral blood (93). As indicated above (**Figure 3A**; **Tables 3**, **S1**) the sources of the MSCs in these studies were remarkably heterogeneous, with many additional variables during their manufacturing. The sources included MSCs from BM, from AT, and from various PT sources, such as from UC-derived Wharton’s Jelly, from Wharton’s Jelly plus selection for ACE2-negative cells, from the subepithelial layer of the UC after discarding the Wharton’s Jelly, from the fetal placenta, from the decidua (maternal side) of the placenta, from menstrual blood, but also MSCs derived from an embryonic cell line, and stromal cells isolated from heart tissue. The closest to a uniform group of cell types is the 11 trials that employed MSCs from Wharton’s Jelly alone without further selection.

We have summarized the cell product manufacturing for all 26 published trials in **Table 4**, while **Figure 3B** summarizes parameters for the 24 published studies on intravenous MSC therapy. Some trials employed more than one MSC product with different processing, such as fresh placenta MSCs and frozen UC MSCs (**Table 3**), so that for some parameters in 3B the total exceeds n=24. The 24 published studies that employed MSCs relied mainly on cell isolation by explants in nine trials (**Figure 3B4** and **Table 4**), by enzymatic digestion in five trials, one trial combined MSCs isolated by each method, one trial alternated between MSCs isolated by each method, and the cell isolation method in the other eight MSC trials was unknown. The three studies that started with blood (either peripheral or menstrual blood), used centrifugation as their first step towards cell isolation. In the MSC trials, the cell expansion/culture process was monolayer in 14 trials (**Figure 3B5** and **Table 4**), only one MSC trial employed a bioreactor, and not stated in nine studies. The number of passages in MSC products was reported for 18 trials and ranged from P3 to P12 with a median of P4 (**Figure 3B6** and **Table 4**), while passages were unknown for six MSC trials. The medium used to grow MSCs for human clinical application contained fetal bovine serum (FBS) in seven of the products (**Figure 3B7** and **Table 4**), while xeno-free medium was used in eight MSC products, and the medium formulation was unknown in nine products. Considering their storage and clinical delivery, 15 of the reported MSC trials used a previously frozen product readily derived from prior cryostorage (**Figure 3B8** and **Table 4**). Two MSC trials used cells fresh from culture, one trial alternated between fresh or frozen MSC products, and in six trials the storage was not reported. The 24 published MSC trials all delivered MSCs by intravenous (IV) route of administration (**Table 3**). The cell dose was scaled by patient weight in 14 of the published clinical trials but set at a fixed dose in the remaining ten studies (**Figure 3B9**). For a patient weighing 70kg, the average cumulative MSC dose across all the trials was 225 million cells per patient, ranging from a minimum of 70 million to a maximum of 630 million cells per patient, thus typically 1-10 million cells/kg of patient body weight, which is the most commonly reported dose range in clinical trials involving IV delivery of MSCs (2, 16–18). Considering patient enrollment, despite the difficulty accruing patients for cell therapy trials during a pandemic with moving surges, we found that five of the 26 published trials managed to accrue more patients than the target enrollment listed in their trial registration (**Table 5**). The average target enrollment was 40 patients and the average achieved enrollment was 29 patients.

**Table 5:** Survival Outcomes of Published COVID-19 MSC Therapy Trials.

Published Studies MSC IV Therapy for COVID-19	Study Design	Number MSC Patients	Number Control Patients	MSC Survival (%)	Control Survival (%)	Study End Point
**Adas et al., 2021 (** 37 **)**	Randomized controlled	10	20	70%	70%	Survival in ICU
**Aghayan et al., 2022 (** 38 **)**	Randomized placebo controlled	10	10	50%	50%	Survival 28 days
**Dilogo et al., 2021 (** 39 **)**	Randomized controlled	20	20	50%	20%	Survival 40+ days
**Farkhad et al., 2022 (** 40 **)**	Non-randomized placebo-controlled	10	10	80%	90%	Survival 17 days
**Gregoire et al., 2022 (** 41 **)**	Controlled ^a^	8	24	100%	79%	Survival 28 days
**Häberle et al., 2021 (** 42 **)**	Placebo-controlled	5	18	80%	44%	Survival in ICU
**Karyana et al., 2022 (** 43 **)**	Randomized Placebo-controlled	6	3	100%	100%	Survival 28 days
**Lanzoni et al., 2021 (** 44 **)**	Randomized controlled	12	12	83%	42%	Survival 28 days
**Leng et al., 2020 (** 45 **)**	Placebo-controlled	7	3	100%	67%	Survival 14 days
**Meng et al., 2020 (** 46 **)**	Controlled	9	9	100%	100%	Discharge from Hospital
**Monsel et al., 2022 (** 47 **)**	Randomized Placebo-controlled	21	24	76%	83%	Survival 28 days
**Rebelatto et al., 2022 (** 48 **)**	Randomized placebo-controlled	11	6	55%	83%	Cytokine markers 4 months
**Shi et al., 2021 (** 49 **)**	Randomized Placebo-controlled ^b^	35	65	100%	100%	Decrease in Lung Lesions
**Shu et al., 2020 (** 50 **)**	Randomized controlled	12	29	100%	90%	Survival 28 days
**Singh et al., 2020 (** 51 **)**	Controlled ^a^	6	34	100%	82%	Discharge from Hospital
**Xu et al., 2021 (** 52 **)**	Placebo-controlled	26	18	92%	67%	Survival
**Zhu et al., 2021 (** 53 **)**	Randomized Placebo-controlled	29	29	100%	93%	Survival 28 days
**Feng et al., 2020 (** 54 **)**	Single arm	16	0	88%	n/a	Survival 28 days
**Hashemian et al., 2021 (** 55 **)**	Single arm	11	0	55%	n/a	Survival
**Iglesias et al., 2021 (** 56 **)**	Single arm	5	0	60%	n/a	Discharge from Hospital
**Sadeghi et al., 2021 (** 57 **)**	Single-arm ^c^	9	0	89%	n/a	Discharge from Hospital
**Saleh et al., 2021 (** 58 **)**	Single arm	5	0	100%	n/a	Survival 28 days
**Sanchez-Guijo et al., 2020 (** 59 **)**	Single arm ^d^	12	0	92%	n/a	Survival in ICU
**Sharma et al., 2022 (** 60 **)**	Single arm	10	0	100%	n/a	Discharge from Hospital

Summary of study design and survival outcomes in 24 publications from completed MSC therapy trials for COVID-19 registered Jan. 2020 to Dec. 2021 and published by the end of July 2022. Publications are sorted alphabetically according to the first author in two groups: first all studies with controls, then all single arm studies. ^a^Control group is retrospective, ^b^Most patients were convalescent, ^c^Excludes a patient who left against medical advice, and ^d^Excludes a patient that died of bleeding caused by a nasal-gastric tube. COVID-19, coronavirus-induced disease 2019; MSC, mesenchymal stromal/stem cells; ICU, intensive care unit; IV, intravenous.

### 3.4 Published clinical trial outcomes: safety and efficacy based on RR/OR analysis

Two previous studies that connected advanced cell therapy clinical trials with their reported outcomes both found that only about 20% of these trials get published eventually (23, 96). To anticipate how many more publications of cell therapy for COVID-19 may be in preparation, we checked the status of all 195 of the 2020 and 2021 clinical trials, as of July 2022. We found that 27 (14%) of the trials had notifications that they had been cancelled, withdrawn, or terminated early. When an explanation was given for these premature endings, typical reasons stated were a lack of funding, or the inability to recruit patients. In addition to the 26 trials (13%) that have been published already, we found another 28 trials (14%) recorded as “completed”, which means that additional peer reviewed publications of cell therapy trials for COVID-19 can be anticipated (final publication rate 27%). Most of the 26 trials published so far were controlled studies: 11 were randomized controlled trials, seven trials included a control group without randomization, and eight trials had a single arm (**Table 5**).

**Figure 4:**
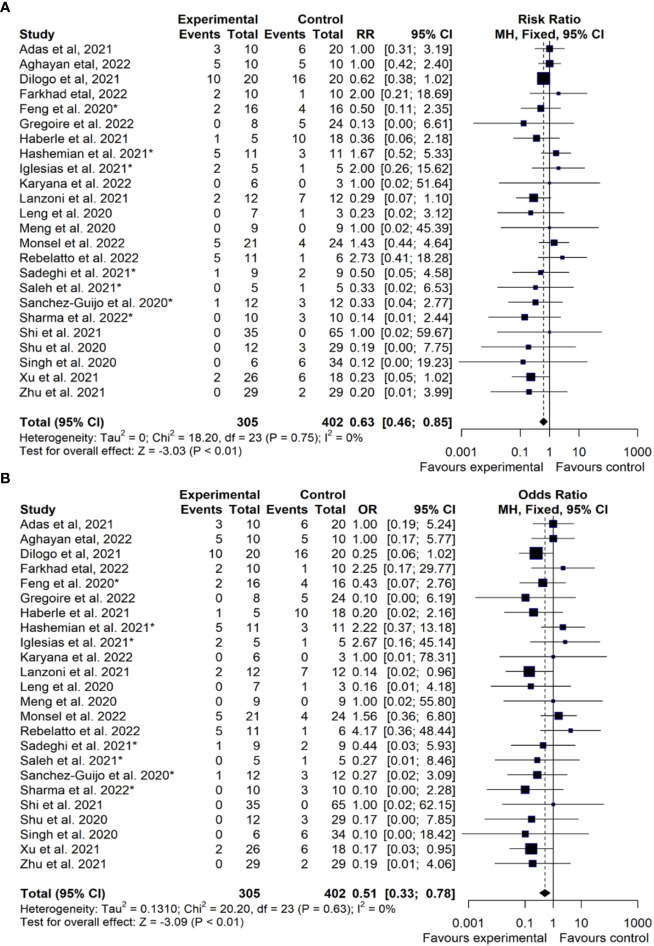
Treatment Efficacy of MSC Therapy for COVID-19 (RR/OR Analysis). Comparison of efficacy of mesenchymal stromal/stem cell (MSC) therapy (Experimental) vs. standard of care (Control), depicting calculations of: **(A)** Risk Ratio (RR) or **(B)** Odds Ratio (OR) for published cell therapy trials employing different types of MSC products (n=24 studies). This analysis includes MSC trials registered in the time period Jan-2020 to Dec-2021, with a follow-up period until the end of July 2022, to also detect trials published after the primary time window. The publications are sorted alphabetically according to the first author. CI, confidence interval; The asterisks (*) indicate the n=7 studies (54–60), where the values for missing controls were computed as indicated in more detail in the methods section. For the double-arm-zero-event studies (43, 46, 49) we employed a treatment arm continuity correction (TACC) to incorporate them, since otherwise RR/OR could not have been calculated (61, 62).

We computed a meta-analysis of the survival benefit from IV MSC therapy for COVID-19. As explained above, the Relative Risk (RR) represents the ratio of the treated patients divided by the whole population (**Figure 4A**), while the Odds Ratio (OR) represents the ratio of the treated patients divided by the control group (**Figure 4B**). For the 24 published trials that employed IV MSCs, we used the survival data tabulated in **Table 5** to assess the clinical efficacy of the treatment relative to controls, according to the statistical procedures described in the Methods. Initially we calculated RR and OR for all 24 studies, employing the missing data compensation described in the Methods. With this approach, there are 305 patients in the MSC treatment groups and 402 in the control groups, with 46 and 90 events (mortality), respectively. In the meta-analysis of these 24 studies, MSC therapy was associated with a diminished risk of all-cause mortality RR=0.63 [95% CI 0.46 to 0.85] (P < 0.01) or OR=0.51 [95% CI 0.33; 0.78] (P <0.01). We repeated our RR and OR calculation using only the 17 of 24 IV MSC studies that had a control arm (**Figure S1**). The existence of control arms means it is not necessary to perform any statistical procedures to correct for missing data. This group had a total of 237 patients in the MSC treatment group and 334 in the control group, with 35 and 73 events (mortality) reported, respectively. Here, MSC therapy was associated with a diminished risk of all-cause mortality RR=0.62 [95% CI 0.45 to 0.87], (P < 0.01) (**Figure S1A**) or OR=0.48 [95% CI 0.29 to 0.81] (P < 0.01) (**Figure S1B**). It is reassuring that the statistical results for this sub-group are almost indistinguishable from the RR and OR results of the full set of 24 studies (**Table 6**).

**Table 6:** Summary of Reported RR/OR in Meta-Analyses of MSC Trials for COVID-19.

Meta-Analysis Study (Author, Year)	No of Studies Included in Meta-Analysis	Risk Ratio [95% CI]	Odds Ratio [95% CI]
**Qu et al., 2022 (** 97 **)**	N=10	0.54 [0.35; 0.85]	—
**Kirkham et al., 2022 (** 98 **)**	N=9	0.50 [0.34; 0.75]	—
**Zhang et al., 2022 (** 99 **)**	N=12 (N=11 MSCs)	—	0.24 [0.13; 0.45]
**Taufiq et al., 2023 (** 100 **)**	N=06	0.65 [0.44; 0.96]	—
Couto et al., 2023* **All MSC studies including missing controls**	**N=24**	**0.63 [0.46; 0.85]**	**0.51 [0.33; 0.78]**
**Couto et al., 2023** **MSCs only controlled studies**	N=17	0.62 [0.44; 0.87]	0.48 [0.29; 0.79]
Couto et al., 2023 * **Perinatal MSCs** **including missing controls**	N=18	0.75 [0.54; 1.02]	0.64 [0.40; 1.03]
**Couto et al., 2023** **Perinatal MSCs** **only controlled studies**	N=12	0.75 [0.53; 1.07]	0.63 [0.36; 1.11]
Couto et al., 2023 * **Non-Perinatal MSCs including missing controls**	N=06	0.27 [0.10; 0.69]	0.19 [0.06; 0.57]

Meta-Analyses of Risk Ratio (RR) and Odds Ratio (OR) for all-cause mortality when MSCs are administered intravenously to treat COVID-19. In this paper (Couto et al., 2023) the calculation is performed for several sub-groups of the 24 articles published so far. Our results are compared to previous meta-analyses of MSC infusions for COVID-19. Our* represents the second approach in this manuscript, which used reconstructed data where the control group was missing. COVID-19, coronavirus-induced disease 2019; CI, Confidence Interval; MSC, mesenchymal stromal/stem cells. The main result of this study are written in bold.

We have summarized the RR and OR meta-analyses for different sub-groups and compared them with previous reports that presented RR/OR survival benefit of cell therapy for COVID-19 (**Table 6**) (97–100). The first two previous meta-analyses in our table only used studies of IV MSC against controls, finding RR=0.54 for ten studies (97) and RR=0.50 for nine studies (98), respectively. While these two meta-analyses had very similar results, their statistical methods differed slightly. The first one included studies with no mortalities on either arm, whereas the second study excluded them. We have included studies with no mortalities by assigning them RR=1.0. A third previous meta-analysis found OR=0.24 for twelve studies (99), although we caution that their meta-analysis mixed different cell types in the statistics.

## 5 Discussion

To the best of our knowledge, this effort is the first report to date, that comprehensively links clinical trials of advanced cell therapy for COVID-19 with the published outcomes of those trials. This type of linkage requires that the starting database of clinical trials is as complete as possible but avoids/omits any redundancies. Thus, in the process of building the COVID-19 trials database at CellTrials.org, we have incorporated several crucial quality steps, e.g. inclusion of trials from all national registries, exclusion of false positives on keywords, and exclusion of double counting of the same trial. So far, none of the other existing trial compilations that we examined in **Table 2** stated that they have employed such steps. We also must point out, that most academic studies of COVID-19 cell therapy trials ran their entire search at a single point in time and selected only for COVID-19 trials. In contrast, CellTrials.org has collected all advanced cell therapy trials monthly and then extracted the COVID-19 trials at the end of each month. In a typical month, CellTrials.org sorts through about 600 clinical trials that hit on keywords and finds that 10% qualify as advanced cell therapy trials.

The advanced cell therapy trials for COVID-19 have been conducted in 30 countries led by the US, China, Iran, and Spain, yet most resulting publications have come from China, Iran, and 11 other countries so far. The initial surge in clinical trials registered to apply cell-based therapy for treating COVID-19 peaked in April 2020 and subsided into an ongoing effort of a few new trials per month. It must be noted that many healthcare policies at both national and local levels influence the ability to launch trials and recruit COVID-19 patients for cell-based therapies. Despite ongoing outbreaks in the US, we have noted that multiple trials have been suspended because they cannot recruit enough patients at a single hospital. Thus, large research consortia with multi-institutional and multi-national collaboration are needed to tackle this shortcoming and more rapidly develop new treatment approaches for COVID-19. In China, the “Zero COVID” policy was so efficient at suppressing outbreaks for two years, that clinical trials had stopped because they could not accrue patients (54). Since this policy was changed in late 2022, new infections and probably also associated severe cases and deaths due COVID-19 are likely to have surged dramatically (101), with a need for effective treatments.

In many Western countries (e.g. Europe being subject to both national and EMA regulation, with considerable variability in regulation between different European nations) (2, 24), strict regulations on human cell therapy mean there were only a limited number of cell therapy products with established safety profiles that could be trialed. While we found that 56% of the trials were below phase 2, we found that 69% of the published outcomes were studies with a control arm. The fraction of the trials employing MSC was 72%, but 92% of the published outcomes are studies that relied on MSCs. The mechanisms of action by which ARDS and COVID-19 patients may benefit from MSC therapy have been exhaustively reviewed by the papers listed in **Tables 2**, **3** (37–53, 63, 64, 66–86, 97–100), such as relying on multiple synergistic effector mechanisms, and promoting/triggering beneficial immunomodulatory and regenerative pathways, as well as angiogenesis and antiapoptosis (18). Hence, we will not repeat that discussion here, but only refer to the most crucial key observations in the discussion further below. Most importantly, in this study, we have also endeavored to quantitatively calculate the safety and efficacy profile of MSC infusions as a novel treatment for COVID-19. However, efforts to treat these topics systematically appear to be fraught with difficulties.

On the issue of MSC safety, we found that all of the 24 published trials claimed that they had no severe adverse events related to the MSC infusion (37–60). Many of the studies gave anti-coagulant therapy as a prophylaxis (38, 42, 44, 56, 57, 60). Already in the first reports from the COVID-19 epicenter in Wuhan, and swiftly following global reports, severe coagulopathy was identified as one of the most evident complications arising from SARS-CoV2 infection and critical/severe COVID-19 (102–112). The increased incidence of thrombotic complications in these patients was verified in large population studies in Sweden (110, 111). Indeed, MSC-IV therapeutics carry a risk of thrombotic complications, due to their expression of the highly prothrombotic tissue factor (TF/CD142) (2, 16, 17). Hence, since the first months of the pandemic, we have identified prophylaxis against coagulation as a crucial factor for the safe application of IV MSC therapeutics in a COVID-19 setting (2, 14, 15). We were poised to highlight the safety aspect of IV MSC therapy for COVID-19, but we discovered that none of the published trials had conducted monitoring that could address this issue. For example, in one trial that treated patients suffering from severe COVID-19 in a hospital ICU, a patient that received IV MSC therapy died 13 days later following an arterial thrombosis (56). This event was considered unrelated to MSC therapy because it occurred outside their four-hour window of post-infusion monitoring.

This was common in the 24 publish trials: possible complications arising from MSC infusion were only monitored during or shortly after infusion. Yet, case studies of adverse events associated with MSC infusions found that elevated clotting markers (e.g. TAT and D-Dimer) typically peaked 9-12 hours post-MSC infusion (17), while pulmonary (but not arterial) embolism post MSC therapy could be detected days, weeks, and even months after treatment (17, 113, 114). This should caution us that the impact of the instant blood-mediated inflammatory reaction (IBMIR) post IV MSC therapy for COVID-19 may only become evident at later time points (2, 16, 17). Given that thromboembolism is a well-known side effect of either COVID-19 infection or MSC infusions, it is currently not possible to retrospectively assign that complication to one or the other group (17), when these studies were not constructed to discriminate the potential causes. Thus, we did not attempt to compute the rate of adverse events in the published trials because the few observed/reported events are largely anecdotal in nature and cannot be quantified at the current stage. Hopefully, future clinical trials will incorporate longer monitoring periods and will have large enough patient groups to statistically identify if any adverse events were increased in response to the experimental (MSC) treatment but not in response to the standard of care.

The primary theme of our analysis was to calculate the efficacy of MSC infusions as a therapy for COVID-19 based on the risk of mortality. The mortality data represent a quantitative set of facts that were extracted from each of the published clinical trials and are listed in **Table 5**. However, we must caution that not all these trials were designed with survival as an endpoint. For example, one study recruited convalescent patients to determine if MSC infusions would speed up the resolution of their lung lesions and consequently none of the patients died on either arm (49). The previous meta-analyses have also noted that published studies with intravenous use of MSC for COVID-19 have very heterogeneous patient populations, in terms of the severity of their COVID-19 illness as well as the forms of concomitant therapies that they received (97–100). Our statistical model has included more studies and more patients than previous meta-analyses of MSC efficacy for COVID-19 (97–100). We corroborate the conclusions of the previous meta-analyses, that intravenous MSC therapy appears to provide a benefit for the treatment of COVID-19 (97–100). Recently, earlier timing of MSC infusion has emerged as a new factor that may be associated with improved survival for patients receiving MSC therapy for COVID-19 (115). This was established in a single center study that gave an IV MSC dose 3 x 3 million cells/kg. We note that their cumulative dose is about three times higher than the average in our 24 studies of IV infused MSCs. We do find that most published studies (17 out of 24) spread out clinical MSC delivery over two to four doses.

Perinatal sources of MSCs (e.g. tissue of UC and/or placenta, collectively called PT-MSCs) (16, 17), were employed in 18 of 24 published trials (**Table 3**). Despite a great deal of preclinical and clinical research, there are still conflicting opinions on the biological characteristics of MSCs isolated from perinatal *versus* adult sources. Multiple earlier studies suggested that PT-MSCs may have superior immunomodulatory properties (116–123), higher proliferation (117, 124), and richer secretome (120, 125–127), compared to adult sources. In turn, some studies also reported that adult sources of MSCs have superior or similar immunomodulatory ability (126, 128, 129), and that adult MSCs have higher production rates of several vital molecular mediators [e.g. VEGF (126, 127), PLGF (126), IL-10 (130), and TGF-β1 (130)] when compared to perinatal cells. Intriguingly, when we performed meta-analysis for subgroups of our data, we found that the RR of the six studies with non-perinatal MSCs was more than factor two better than the RR of the 18 perinatal studies (**Table 6**). All six of the non-perinatal studies, which employed five different MSC products, reported good outcomes. In contrast, among the 18 perinatal studies there were five studies where mortality on the MSC treatment arm was higher than the control baseline (40, 47, 48, 55, 56). However, this RR comparison was not statistically significant, leading us to argue that more data is needed to confirm or refute this result.

Another focus of our study was to evaluate the diversity of manufacturing methods used to prepare MSC products and to highlight the importance of reporting manufacturing information to enable study comparability (**Figure 3**; **Tables 3**, **4**). Indeed, diversity in cell product manufacturing parameters, cell dosing, and cell characterization for therapeutic use, but also the completeness of study descriptors, have all been identified as a potential confounder to interpretation of safety and efficacy outcomes in MSC studies and should be monitored/reported more thoroughly in future studies (2, 16, 17, 33, 131–133). Recent reviews (131, 132) have highlighted the frequent lack in reporting of MSC manufacturing and study descriptors as a considerable shortcoming to clinical trial reporting and subsequent study interpretation. This aspect is of such importance, that it has been taken up into: “A modified Delphi Study Protocol” for “Establishment of a Consensus Definition for Mesenchymal Stromal Cells (MSC) and Reporting Guidelines for Clinical Trials of MSC Therapy” (133). For example, one multi-center randomized control trial (RCT) that employed UC-MSCs to treat COVID-19 found that MSC therapy had no efficacy (47). However, the MSCs in that study were manufactured in a laboratory that alternated between cell isolation with explants vs. enzymatic digestion (which may impact cell yield and immunophenotype) (134, 135). Still, the research consortium did not publish any records of which patient received which MSC product (47). Consequently, the outcome of the entire multi-center RCT may be cast into doubt because it is unknown how manufacturing variability may have compromised the respective results. Another manufacturing issue, which has been largely overlooked in the discussion of cell therapy trials for COVID-19 so far, is the ability to scale up the production of successful therapies (31, 136–139). Most trials published so far have relied on 2D monolayer cell expansion methods in flasks, with only one study employing a hollow-fiber bioreactor (42). The monolayer methods are time-consuming, labor intensive, and have limited scalability (135, 140, 141). Given the potential combinatorial detrimental impact of both freeze-thawing and *in vitro* aging on cell potency (33, 87–91, 135), it is of importance to accurately evaluate the number of population doublings a cell product has accumulated during *ex vivo* culture expansion for COVID-19 therapy and other indications (2, 14, 16). Of course, it is also relevant to record if the cell product was given either fresh or as a freeze-thawed product derived from cryostorage, since this may impact substantially on the product performance (14, 30, 31, 33, 88–91, 142–144).

## 6 Conclusions and limitations

The International Society for Cell and Gene Therapy (ISCT) recently published an editorial calling for a global registry of clinical trials that employ MSCs for COVID-19 to harmonize the data on the limited number of patients and “To collect information on critical process parameters used to manufacture the MSCs” (145). We support that call to action. Our review of the manufacturing parameters in clinical trials giving cell-based therapy for COVID-19 has revealed a partial disconnect between clinical centers that treat patients versus laboratories that manufacture cell therapy products. Frequently, the clinicians running the trials have acquired cells and delivered them to patients without keeping any records about the cell production. This disconnect could be closed, if the clinical trials participated in a global registry that required completing standardized categories of information. In this study we have compiled two years of worldwide clinical trials testing cell-based therapies for COVID-19 and linked those trials to their published outcomes. This “end-to-end” survey of the research field has enabled us to learn new insights not published earlier. First, we found that global registrations of advanced cell-based therapies for COVID-19 were more numerous than previously reported, but that they experienced only one single early surge in trial registrations during a time frame, when global COVID-19 infections went through multiple surges. Our analysis also includes the contribution from registered clinical trials that are not listed on the national registries of the United States and China, with 53 and 43 trials, respectively. Hereby, we have learned that Iran (19 trials) is among the three leading nations running advanced cell therapy trials for COVID-19 and the 2^nd^ in publishing trial outcomes. In turn, Israel, Spain, Iran, Australia, and Sweden are leading in relative contributions to COVID-19 cell therapy trials normalized to population size (N=0.641, 0.232, 0,223, 0.194, and 0.192 trials per million inhabitants).

Although 72% of the COVID-19 cell therapy trials employed tissue-derived MSCs, a significant fraction of clinical trials conducted immunotherapy with blood-derived cells. So far, most of the published trials describe infusions of MSCs, and 75% of those employed MSCs derived from perinatal tissue sources. Throughout these studies there is a strong theme of heterogeneity. The patient groups in the clinical trials are heterogeneous, as are the manufacturing methods used to prepare the MSCs. Most importantly, our statistical analysis shows that infusions of MSCs show a clinical benefit for COVID-19 patients. The risk ratio for all-cause mortality is RR=0.62 [95% CI 0.44 to 0.87] for the 17 MSC studies with control arms, and when we compensated for the missing controls and incorporated all 24 MSC studies the result is RR=0.63 [95% CI 0.46 to 0.85]. Early during the COVID-19 pandemic, it emerged that the respiratory distress caused by COVID-19 is a substantially different clinical entity compared to classic ARDS (21). Hence, we cannot assume that the efficacy results obtained here will similarly influence the long-standing quest to improve ARDS mortality. For this answer, we must await the outcomes of large placebo-controlled randomized trials of MSCs for ARDS, such as the REALIST trial in the UK, and the STAT trial in the US, respectively (146, 147). We close with the concern that there may never be enough data to fully explore the efficacy of cell-based therapy against ARDS from COVID-19. Owing to the evolution of the virus to less lethal variants and the rollout of vaccination, it has recently become difficult to accrue patients for clinical trials that treat severe symptoms of COVID-19 infection. The relative impact of MSC product source, MSC dosing, and the timing and type of MSC delivery, etc., may never be fully explored or known within the current setting of COVID-19.

From a public health perspective, the highest goal is to prevent the development of severe or critical COVID-19 through combined effective pandemic countermeasures (**Figure 1A**) (148). In this regard, vaccination is the most valuable tool available. Also, the standard-of-care for the treatment of severe and critical COVID-19 is continuously improving. Although we here found that infusions of MSCs confer a reduction in the risk for all-cause-mortality from COVID-19 in the studies published to date, more research is needed to clarify this point. Nonetheless, there will always be high-risk patients who develop severe or critical COVID-19, and for them the existence of adjunct treatment with advanced cell therapy may be beneficial. The target groups for whom this therapy may provide benefit include the elderly, immunocompromised individuals, cancer patients, and transplant patients (both stem cell transplants and solid organ transplants) as well as patients with kidney failure on dialysis (17, 149, 150). More research on the efficacy of advanced cell therapy for COVID-19 will reveal the degree to which these groups may benefit. Eventually, the cost and access to advanced cell therapy must also be anticipated, typically requiring advanced medical infrastructure. It is our hope that the testing of advanced therapies will be pursued in parallel to the improvement of standard care.

## Data availability statement

The original contributions presented in the study are included in the article/**Supplementary Material**. Further inquiries can be directed to the corresponding authors.

## Author contributions

PC and GM and FV conceived and designed this study. PC and AB and FV conducted primary data collection. PC and NA-A and IF and DF and RC and OC-M and GM and FV conducted the primary data analysis. PC and GM and FV wrote the first draft of the article. IH and RAC and OC-M. complemented and revised the manuscript and provided resources to support the study. All authors contributed to the article and approved the submitted version.

## Supplementary material

Supplementary Figure 1 Treatment Efficacy of MSC Therapy for COVID-19 (RR/OR Analysis) controlled studies only (Analysis Supplemental to Part A).
