# Supplementary figures and images for: Systematic review and meta-analysis of cell therapy for COVID-19: global clinical trial landscape, published safety/efficacy outcomes, cell product manufacturing and clinical delivery
Source: Front Immunol. 2023 Jun 21;14:1200180. doi: 10.3389/fimmu.2023.1200180 (PMC10321603; doi:10.3389/fimmu.2023.1200180)

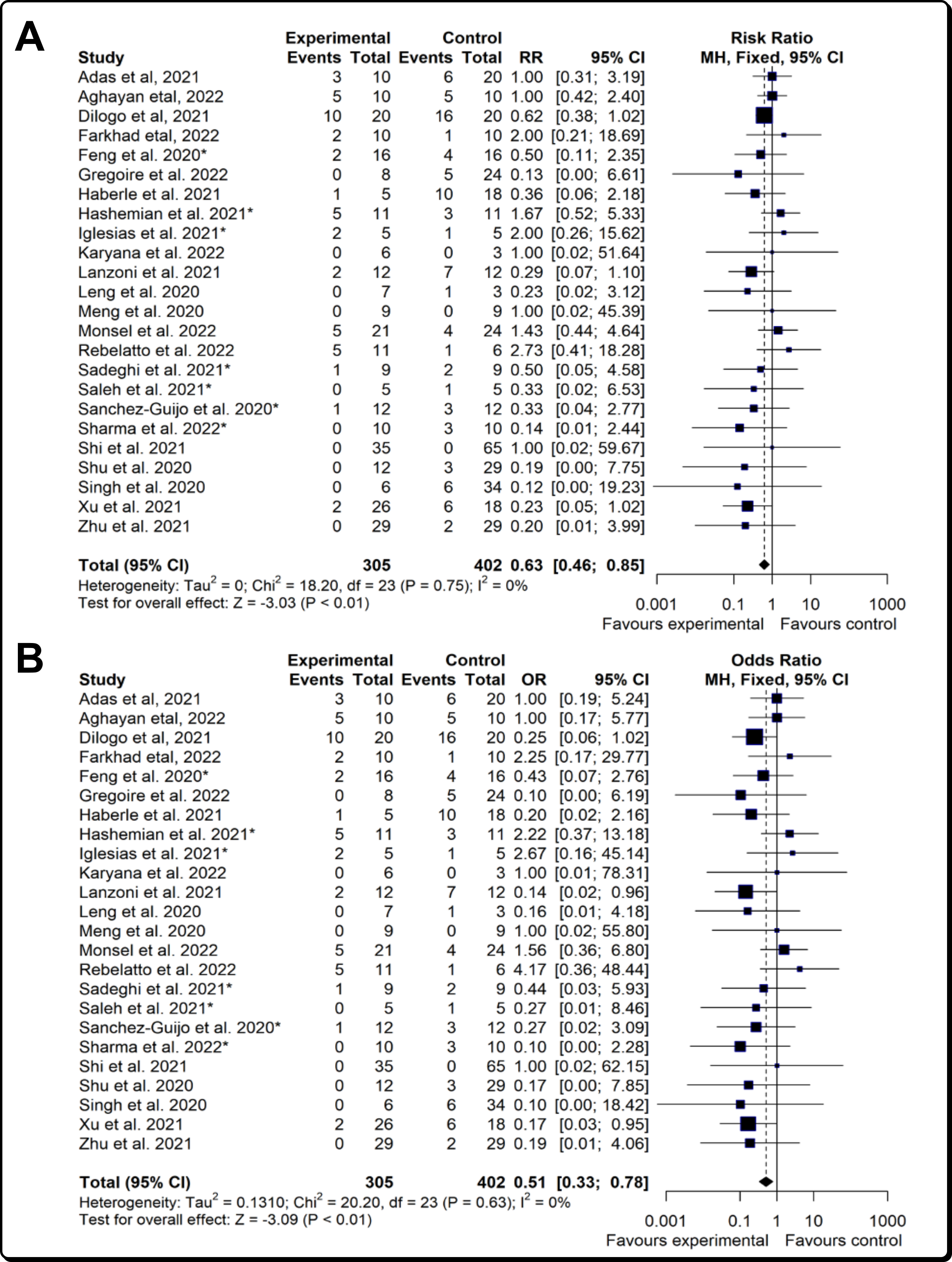

Supplement: Supplementary Figure 1 — Treatment Efficacy of MSC Therapy for COVID-19 (RR/OR Analysis) controlled studies only (Analysis Supplemental to Part A). [file Image_1.jpeg]
